# Common Nettle (*Urtica dioica* L.) as an Active Filler of Natural Rubber Biocomposites

**DOI:** 10.3390/ma14071616

**Published:** 2021-03-26

**Authors:** Marcin Masłowski, Andrii Aleksieiev, Justyna Miedzianowska, Krzysztof Strzelec

**Affiliations:** Institute of Polymer & Dye Technology, Lodz University of Technology, Stefanowskiego 12/16, 90-924 Lodz, Poland; andrii.aleksieiev@dokt.p.lodz.pl (A.A.); justyna.miedzianowska@edu.p.lodz.pl (J.M.); krzysztof.strzeles@p.lodz.pl (K.S.)

**Keywords:** natural rubber, common nettle, seeds, leaves, branches, roots, biocomposites, elastomers

## Abstract

Common nettle (*Urtíca Dióica* L.), as a natural fibrous filler, may be part of the global trend of producing biocomposites with the addition of substances of plant origin. The aim of the work was to investigate and explain the effectiveness of common nettle as a source of active functional compounds for the modification of elastomer composites based on natural rubber. The conducted studies constitute a scientific novelty in the field of polymer technology, as there is no research on the physico-chemical characteristics of nettle bio-components and vulcanizates filled with them. Separation and mechanical modification of seeds, leaves, branches and roots of dried nettle were carried out. Characterization of the ground plant particles was performed using goniometric measurements (contact angle), Fourier transmission infrared spectroscopy (FTIR), themogravimetric analysis (TGA) and scanning electron microscopy (SEM). The obtained natural rubber composites with different bio-filler content were also tested in terms of rheological, static and dynamic mechanical properties, cross-linking density, color change and resistance to simulated aging processes. Composites with the addition of a filler obtained from nettle roots and stems showed the highest mechanical strength. For the sample containing leaves and branches, an increase in resistance to simulated ultraviolet and thermo-oxidative aging processes was observed. This phenomenon can be attributed to the activity of ingredients with high antioxidant potential contained in the plant.

## 1. Introduction

Several advantages resulting from the properties of polymeric materials, such as high chemical and thermal resistance, high mechanical strength, barrier properties [1,2,3,4,5] with the simultaneous ease of processing and forming and a lightness of the products has led to the increasing use of plastics and rubber. As the environmental problems associated with the use of non-renewable polymers sources have not been eliminated, many scientists have focused on alternative solutions that can provide materials with similar properties, but of natural origin [6,7,8]. These works are aimed at full or partial replacement synthetic materials with natural ones and consequently reducing the use of the production of plastics or rubber. Current studies provide knowledge about the influence of various natural components on the properties of the obtained composites [8,9,10].

One of the most popular classes of composite materials with a reduced amount of components from non-renewable sources are biocomposites, which, according to the requirements of green chemistry, should be made of at least one component derived from renewable resources and/or from recycling. Toxic by-products cannot arise during production and modification processes, as well as at any stage of their life cycle or during neutralization and storage [10,11,12,13]. The matrices of biocomposites can be made of petroleum-based polymers or bio-based polymers while the reinforcement is a natural additive such as wood flour, rice husks, coffee husks, cereal straw, hemp, bamboo, etc. [8,9,14,15,16,17,18,19]. The most commonly used materials for biocomposites are thermoplastics and thermoset resins including polypropylene (PP), polystyrene (PS), polyethylene (PE), polyvinylchloride (PVC), epoxy resin, vinyl esters, polyesters and phenol-formaldehyde [20]. Moreover, attempts have been made to create synthetic rubber-thermoplastic blends reinforced with natural filler [21,22,23]. In this study, authors focused on trends in the use of natural elastomers in the production of biocomposites in order to limit synthetic rubbers [8,9,24,25,26].

Among the elastomer matrices, natural rubber can be distinguished, which is the only one that is completely natural high-molecular material. Natural rubber (NR) is obtained from tropical trees and plants rich in latex or milky colloidal suspension. Natural rubber mixtures are water-resistant, have good electrical insulation, low internal friction and resistance to most salts and alkalis [27]. NR vulcanizates can be used as textile cords or coated fabrics, conveyor belts, electric insulation, cables, elastic bands, belts, tires, hoses, gaskets, containers and many more. Due to the wide use of natural rubber, new combinations of this elastomer with various fillers are made to obtain new types of biocomposites [10,13].

Natural fibrous fillers have attracted interest especially due to their low cost and renewability, but also relatively good properties such as low density, their capacity for damping acoustic waves, biodegradability and the ease of recycling [28]. The fibrous structure contributes to the increase in mechanical strength, barrier and damping properties of biocomposites [8,29]. The most valuable fibrous fillers are those growing wild as weeds or being waste, which can determine a filling with low sourcing costs to obtain a polymer composite. Another advantage of using natural fillers is the possibility of minimizing the number of toxic additives (e.g., cross-linker) necessary to create a natural composite. In order to obtain a durable fiber based biocomposite, fibers often need to be modified to ensure the best possible connection between the hydrophilic filler and the hydrophobic polymer matrix [30,31,32,33]. The greatest problem in the use of natural fibers is their hygroscopicity and the high variability of properties which depend on factors such as fiber type and its modification, environmental conditions or processing methods. In order to overcome them, various approaches have been taken, including chemical and physical treatment [19,26]. Natural fiber-reinforced biocomposites have been used for many applications, such as automobiles, aerospace, packaging and building industries, foams, coatings, printing inks, lubricants, plastics, etc. [11]. The most commonly known composites filled with natural fibers are wood composites including plywood, fiberboard, chipboard, glulam, cross-laminated timber, etc. [34]

Common nettle (*Urtica dioica* L.) grows wild in mild to temperate climates especially in forests and shady humid places in Europe, Asia, North Africa and North America. It is considered a weed due to its rapid growth [35]. Common nettle is a plant known primarily for its antioxidant, antiplatelet, hypoglycaemic and hypocholesterolemic properties [36,37]. *Urtica dioica* L. is rich in different ingredients depending on the part of the plant. The leaves are rich sources of terpenoids, carotenoids and fatty acids, as well as of various essential amino acids, chlorophyll, vitamins, tannins, carbohydrates, sterols, polysaccharides, isolectins and minerals, while the roots contain oleanol acid, sterols and steryl glycosides [35]. Nettles are used in traditional medicine and the food industry [38]. The analysis of the literature shows that attempts were made to create biocomposites reinforced with common nettle [39,40,41] as well as a modification to improve compatibility between hydrophilic natural fiber and hydrophobic polymer matrix in the form of chemical treatment with NaOH [42].

The aim of this work was to investigate the activity of natural filler in the form of common nettle in the obtained biocomposites. The scientific literature does not deliver a detailed description of mechanical, rheological and thermal properties of the natural rubber biocomposite with the addition of *Urtica dioica* L. This research as a scientific novelty will allow to determine the usefulness of the selected plant in natural rubber technology.

## 2. Materials and Methods

### 2.1. Materials

- Elastomer matrix: natural rubber RSS I (NR) provided by Torimex Chemicals (Konstantynów Łódzki, Poland).
- Conventional sulphur curing system consisting of: sulphur (S) (Siarkopol, Tarnobrzeg, Poland), micro-sized zinc oxide (ZnO) (Huta Będzin, Będzin, Poland), mercaptobenzothiazole (MBT) (Sigma-Aldrich, Poznań, Poland), stearin (POCH, Gliwice, Poland).
- Natural filler: common nettle (*Urtica dioica* L.).

*Urtica dioica* L. used in the research was collected at the end of July, after the rainy season, in an ecologically clean place, such as the shores of a water reservoir in the region of Lodz Province, Poland (51°45′13.3″ N, 19°26′22.8″ E). Before cutting, the quality of the common nettle was visually assessed, especially for the presence of bacterial infections, various parasites and spider webs. The selected plants were tall (approx. 1 m) with wide leaves, thick stems and large roots. The stalks were cut with a knife, and then the separation of seeds, leaves and stalks (hereinafter referred to as branches) was performed. The roots of the cut stems were dug separately, after which they were washed alternately in warm and cold water in a wicker basket. Fine roots, stem remnants and rotten parts were cut off. The raw materials were dried in a forced air dryer (Binder, Tuttlingen, Germany) at 30 °C for 28 days.

The compositions of rubber mixtures are presented in Table 1. Twelve common nettle filled composites and reference sample of unfilled natural rubber were prepared for this study. Constant ratio (%) of constituent materials in CNs for each part by weight (seed, leaves, branches and roots) including the cross-link unit, is respectively: for 10 phr = 8.33%; for 20 phr = 15.39%; for 30 phr = 21.43%. mill

In order to identify prepared samples, the following abbreviations were used: NR—Natural Rubber; CN—Common Nettle; s—seeds; l—leaves; b—branches (stems); r—roots; numbers: 10, 20, 30—the content of filler: 10 phr, 20 phr, 30 phr.

### 2.2. Preparation of Fillers

The preparation of the fillers was started by grinding the seeds, leaves, branches and roots of the common nettle separately with a Fritsch Pulverisette 5 planetary mill (Fritsch, Ilztal, Austria) in 500 mL hardened, stainless steel grinding bowl with twelve 15 mm diameter stainless steel spheres for 40 min at a rotation speed of 300 rpm. Then, the powdered part of the CN was allowed to dry at 35 °C for about 48 h.

Each part of the nettle was then screened through a Model 911M PESM laboratory vibratory sieve machine (911 Metallurgy Corp. Langley, BC, Canada) and the results of this analysis are shown in Table 2.

### 2.3. Preparation of Rubber Mixtures

The preparation of elastomer blends was carried out in the following stages:

- plasticizing of natural rubber by mixing in a Brabender N50 (Brabender Technologie GmBH & Co. KG, Duisburg, Germany) measuring mixer with process parameters: rotational speed 40 rpm, temperature 40–60 °C, mixing time 4 min,
- dispersing fillers in plasticized natural rubber for 4 min in the same conditions,
- introducing a weighed sulphur curing system for obtained mixtures on a two-roll mill at room temperature.

### 2.4. Methods

Thermogravimetric analysis of the dried Common Nettle (seeds, leaves, branches, roots) was performed using the TGA/DSC1 analyzer (Mettler Toledo, Columbus, OH, USA/Greifensee, Switzerland). The thermograms were recorded between 25 and 700 °C with a heating rate of 10 °C min^−1^ in a flow of nitrogen at 60 mL min^−1^. The test was repeated once for each sample.

The FTIR Nicolet 6700 (Thermo Fisher Scientific, Waltham, MA, USA) reflection ATR technique on an adapter with diamond crystals on an ZnSe plate was used to obtain the FTIR spectra. The FTIR spectra of Common Nettle and composites reinforced with 30 phr of the filler were recorded at a resolution of 8 cm^−1^, with 128 scans, over the range of 4000–400 cm^−1^. The test was repeated once for each sample.

Diffuse reflectance UV-Vis spectroscopy was performed on an Evolution 201/220 UV-Visible Spectrophotometer (Thermo Fisher Scientific, Waltham, MA, USA). Measurements of the powdered filler were conducted in the spectral range of 1100–200 nm. The test was repeated once for each sample.

Wetting of separated parts of the common nettle was define by contact angle (Θ) measurement using Dataphysics OCA15EC (DataPhysics Instruments GmbH, Filderstadt, Germany). Powdered materials were compressed in the form of the sample with the smooth surface (tablet). The contact angle was measured by placing a drop of water (1–2 µL) on the tablet. The analysis of measured Θ allows the hydrophilicity or hydrophobicity of the material to be determined. Contact angle below 90° indicates hydrophilic materials and above 90°—hydrophobic [43]. The test was repeated 5 times for each sample.

Rheometric properties of the rubber mixtures were studied at 160 °C using an MonTech DRPA 300 Rheometer (MonTech Werkstoffprüfmaschinen GmbH, Buchen, Germany) following ISO 3417:1994 standards. Each sample was placed in the measuring chamber of the device and subjected to changes in the torque of the oscillating disc as a function of time. The test was repeated once for each sample. The following parameters were determined:

- optimal curing time t_90_ (min), which occurs at 90% increase in torque;
- increase in torque ΔM (dNm) as a difference of maximum (M_max_) and minimum torque (M_min_) during measurement.

Vulcanization process of the mixtures was carried out in a hydraulic press equipped with a mold at 160 °C and 15 MPa for time determined from rheometric measurements.

The thermo-oxidative aging simulation process was carried out in a forced air dryer at 70 °C for 14 days. UV degradation was accomplished by using Atlas UV 2000 (ATLAS Material Testing Technology GmbH, Duisburg, Germany) equipment. The samples of NR filled with CN and reference sample were placed in holders with dimensions of 9.5 cm/6.5 cm and fixed in the UV chamber with the following degradation parameters:

- The day and night segment: 0.78 W/m^2^;
- Temperature: 60 °C;
- Duration: 72 h.

After the simulated aging processes, the vulcanizates were analyzed in terms of mechanical properties and cross-linking density.

The cross-linking density of the vulcanized network on non-aged and aged rubber mixtures was calculated on the basis of solvent-swelling measurements in toluene from the Flory–Rehner Equation (1) [44]. The test was repeated 4 times for each sample:

(1) $$\gamma_{e}\;=\;\frac{\ln\left({1-V}_{r}\right){+V}_{r}+\mu V_{E}^{2}}{V_{0}\left(V_{r}^{\frac{1}{3}}-\frac{V_{r}}{2}\right)}\;$$

where: $\gamma_{e}$—the cross-linking density (mol/cm^3^), V_0_—the molecular volume of solvent (106.7 cm^3^/mol), μ—the Huggins parameter of the NR-solvent interaction calculated from Equation (2):

(2) $${\mu \;=\;\mu }_{0}+\beta \cdot V_{r},$$

where: μ_0_—the parameter connected with non-cross-linked/solvent, β—the constant consideration of the impact of cross-linking on parameter polymer/solvent, natural rubber–toluene interaction factor μ_0_ and β were experimentally (μ_0_ = 0.478, β = 0.228); Vr—the volume fraction of elastomer in the swollen Equation (3):

(3) $$V_{r}\;=\;\frac{1}{{1+Q}_{w}\frac{\rho_{k}}{\rho_{r}}},$$

where: Q_w_—weight of equilibrium swelling, ρ_k_—density of rubber (g/cm^3^) (0.99 g/cm^3^), ρ_r_—density of solvent (g/cm^3^) (0.86 g/cm^3^).

Barrier properties were examined on the basis of through-plane air permeability of composites using manometric method according to the ASTM standard D1434. Tests were carried out by using atmospheric air at room temperature. The test was repeated once for each sample. The air permeability was determined by gas transmission rate (GTR) from the following Equation (4) [45]:

(4) $$\mathrm{GTR}\;=\;\frac{V_{c}}{R\cdot T\cdot P_{u}\cdot A}\cdot \frac{\mathrm{dp}}{\mathrm{dt}},$$

where: V_c_—volume of low-pressure chamber (L), T—temperature (K), P_u_—the gas pressure in the high-pressure chamber (Pa), A—area permeation of gas through the sample (m^2^), dp/dt—pressure changes per unit time (Pa/s), R—gas constant 8.31 × 10^3^ ((L·Pa)/(K·mol)).

The tensile measurements of both non-aged and aged biocomposites were examined on dumbbell-shaped samples by using a static material testing machine Zwick (model 1435, Ulm, Germany) according to ISO-37 at room temperature. The cross-head speed of testing was 500 mm/min. At least four different samples were tested from each rubber biocomposites.

The ageing coefficient (K) was determined as the numerical change in the static mechanical properties of the samples upon UV degradation process and thermo-oxidative aging Equation (5) [46]:

(5) $$K\;=\;\frac{{(\mathrm{TS}\cdot E_{b})}_{\mathrm{after}\;\mathrm{aging}}}{{(\mathrm{TS}\cdot E_{b})}_{\mathrm{before}\;\mathrm{aging}}},$$

where E_b_—elongation at break, TS—tensile strength.

The polymer–filler and filler–filler interactions were estimated based on the dynamic-mechanical analysis by using the ARES G2 Rheometer (TA Instruments, New Castle, DE, USA). The analysis of the strain scanning applied to the vulcanizates in the range between 0.1 and 100% and frequency of 50 Hz with sample deformation rate 10 rad·s^−1^ and force: 5 N at the temperature of 25 °C was carried out. The test was repeated once for each sample. The Payne Effect (∆G^′^) was calculated from Equation (6):

(6) $$∆{G_{\prime }=G_{\prime }}_{\min}\left({\lim10}^{-1}\right)-{G_{\prime }}_{\max}\left(\infty \right),$$

where G′_min_(lim10^−1^)—a storage modulus determined under the deformation of 0.1%; G′_max_(∞)—a storage modulus determined under the maximum deformation.

The morphology of the fillers particles and NR vulcanizates filled with 20 phr of each common nettle part were examined by Scanning Electron Microscopy (SEM) by using Hitachi TM-1000 (Hitachi Ltd., Tokyo, Japan). Composites were cryo-fractured for this study.

The color change of materials before and after UV and thermo-oxidative aging processes was tested in accordance with the PN-EN ISO 105-J01 standard. The measurements were performed using a Konica Minolta CM-3600d spectrophotometer (Sony, Tokyo, Japan). The color change test is based on converting the light reflected from the surface of the test sample to the color perceived by photoreceptors in the human eye. The result is a color described in the CIE-Lab space and as coordinates that enable the determination of the color in the system of three coordinates: L—brightness parameter; a—axis of red—green; b—axis of yellows—blues. The change of color was calculated from Equation (7) [47]:

(7) $${\mathrm{dE}}_{\mathrm{ab}}^{*}\;=\;\sqrt{∆a^{2}+∆b^{2}+∆L^{2}},\;$$

where: Δa, Δb, ΔL—deviation from the color of the reference sample, analogically on the axes a, b and L.

Due to the specificity of conducting the color change test, a measurement points of CIE-Lab on the surface of specimen was performed in several places (ca. 10); then, an average value was presented.

## 3. Results and Discussion

### 3.1. Characterization of Fillers

#### 3.1.1. Fourier Transform Infrared Spectroscopy (FTIR) Analysis

**Figure 1:**
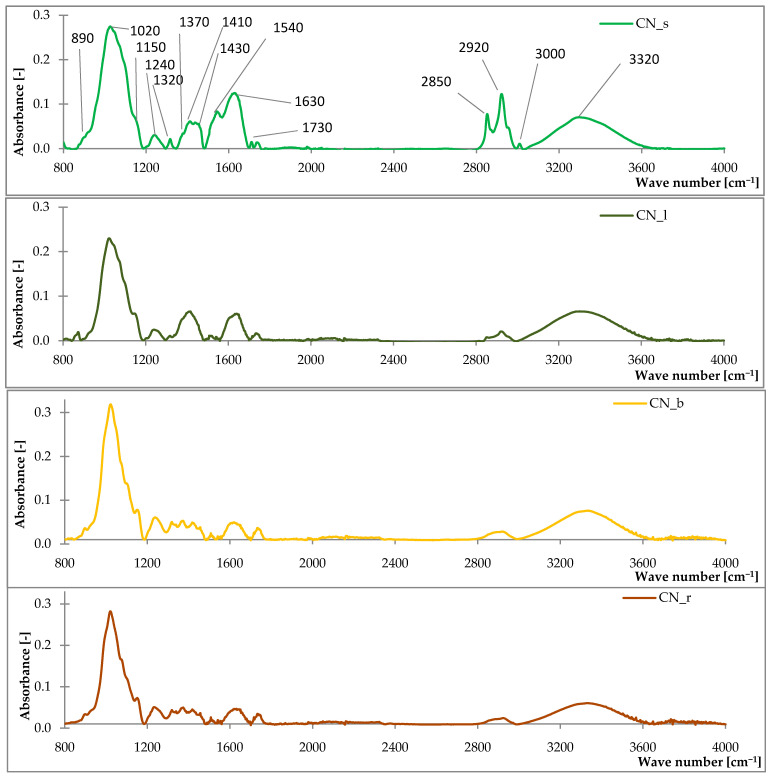
The FTIR spectra of common nettle seeds (CN_s), leaves (CN_l), branches (CN_b), roots (CN_r).

The FTIR spectra of common nettle are shown in Figure 1 and the absorption ranges corresponding to the characteristic bonds are presented in Table 3. A broad band at 3620–3000 cm^−1^ (with a maximum at around 3300 cm^−1^) typical for the vibration of –OH phenols and alcohols groups was presented in the spectra of seeds, leaves, branches and roots of *Urtica dioica* L. These groups occur in the structure of cellulose, hemicellulose and lignin. Peaks at around 3100–2800 cm^−1^ were related to asymmetric and symmetric methyl and methylene stretching groups. Esters and aldehydes groups were noted from the characteristic vibration of C=O in the absorption range of 1730–1690 cm^−1^. Recording at 1750–1500 cm^−1^ absorbance was related to amines, alkenes and aryl groups. A characteristic peaks within the range of 1200–1060 cm^−1^ were typical for the vibration of C–O, C–C, C–O–C groups related to axial deformation, asymmetric and symmetric stretching esters and ethers. The highest absorbance was observed at 1020 related to the asymmetric stretching of phosphates.

The analysis of infrared spectroscopy of the roots, leaves, stems and seeds of the common nettle showed that the plant is composed mainly of lignocellulosic material. This is evidenced by the registered characteristic absorption bands for groups occurring in this type of materials.

#### 3.1.2. Ultraviolet-Visible Spectroscopy (UV-Vis)

**Figure 2:**
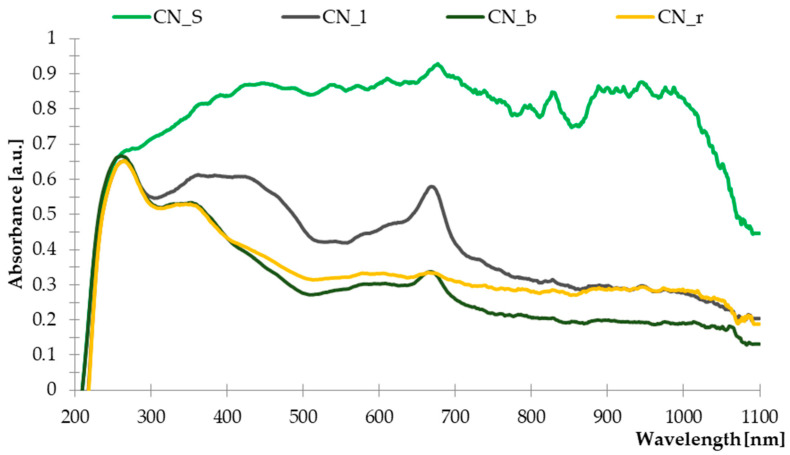
The UV-Vis absorption spectra of common nettle (stems, leaves, roots, seeds).

The UV-Vis spectra of the individual parts of the common nettle are illustrated in Figure 2. The presented results indicate that the materials derived from the common nettle contained dyes with absorption of visible radiation as well as substances capable of absorbing ultraviolet radiation.

When interpreting the obtained curves, it was found that leaves, stems and branches contain chlorophyll. The obtained peaks with the maximum absorption of 680 nm and 380 nm indicated the content of this dye. Chlorophyll is characterized by two absorption regions, similar to other porphyrin derivatives, in the wavelength ranges 380–480 nm and 560–680 nm [48]. Moreover, the band present in the visible region of the spectrum showing an absorption in the range of 400–500 nm indicated the presence of carotenoids in the tested samples [49]. The major carotenoids present in common nettle were lutein, lutein isomers, β-carotene and β-carotene isomers [50]. Moreover, all parts of the plant showed intense radiation absorption bands from 220 to 360 nm. From literature data, it is known that the absorption maximum identified at 360 can be attributed to the presence of flavonol glycosides, while phenolic acids were identified at 280 nm [51,52,53]. These compounds are characterized by high antioxidant activity, hence their anti-aging effect in polymer composites is possible due to their content in the material particles distributed on the surface of the composite.

#### 3.1.3. Thermogravimetric Analysis

**Figure 3:**
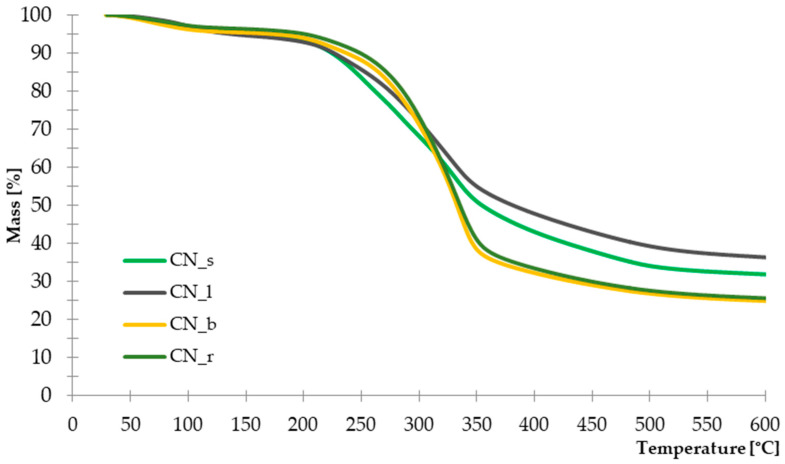
TG curves of Common Nettle seeds, leaves, branches and roots.

**Figure 4:**
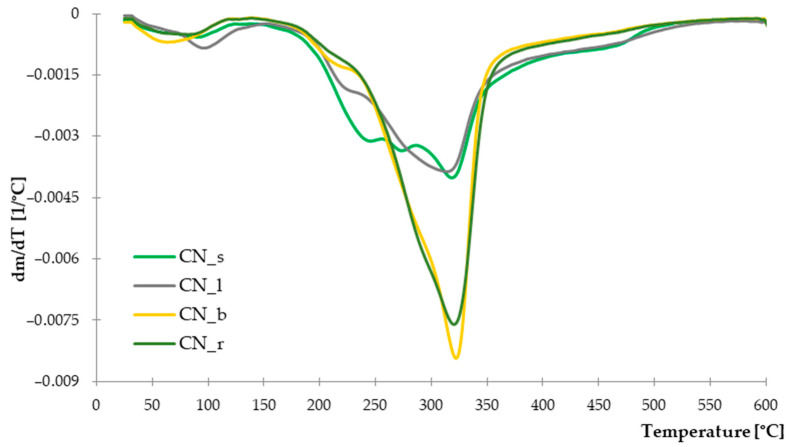
DTG curves of Common Nettle seeds, leaves, branches and roots.

Selected parts of the common nettle (stems, leaves, roots, seeds) were subjected to thermogravimetric analysis in order to determine their thermal stability. The thermal decomposition of individual compounds contained in the plant allows for indicating the temperatures at which the greatest loss of mass occurs. Such information is particularly important from the point of view of biocomposites processing. The recorded TG and DTG curves are presented in Figure 3 and Figure 4. The characteristic parameters of the thermal distribution are presented in Table 4.

The recorded DTG curves show that the thermal decomposition of the tested materials is multi-stage. For all parts of plants tested, the first mass loss occurs in the range of 60–100 °C, with a range of 2.09–3.04%. It is related to the evaporation of water and moisture from the structure of natural fibers. The greatest weight loss for all parts of the nettle is recorded in the temperature range of 170–440 °C. Intensive thermal decomposition of leaves and seeds began at a temperature of about 220 °C (T_10_) and lasted up to 350 °C. However, samples with branches and roots showed a rapid weight loss in the range of 250–330 °C. According to the research on thermal decomposition of natural fibers [54], cellulose degradation is revealed at the temperature of 220–450 °C, while hemicellulose and lignin in the lower range, respectively, 160–300 °C for hemicellulose and 160–400 °C for lignin. The observed differences in the decomposition temperatures of individual parts of the common nettle may indicate a different content of the building chemical components. On the basis of the conducted analysis, it can be assumed that the seeds and leaves contain more thermally less stable lignin and hemicellulose. Hence, on the DTG curve, a distinct, multi-stage course related to the degradation of these compounds was observed. These observations are also confirmed by the recorded values of weight loss at the temperature of 335 °C, in which the leaves and seeds again showed a higher value of Δm_335_. In the case of branches and roots, richer in cellulose, with higher thermal stability, the Δm_335_ value was lower and amounted to 48.13% and 50.39%, respectively (Table 4.).

Moreover, the total residue after pyrolysis at 600 °C for seeds and leaves was 32% and 36%, respectively. This amount was much higher than in the case of nettle branches and roots, where the solid residue constituted approx. 25%. Again, this is confirmed by the higher lignin content of the leaves and seeds as this component is more difficult to decompose and generates much more solid residue [55].

#### 3.1.4. Wettability and Contact Angle

**Figure 5:**
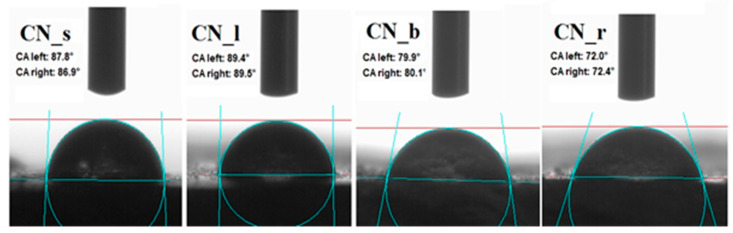
Contact angle (CA) measurements for each part of common nettle.

Measurement of the surface wettability of selected common nettle parts was performed in order to determine the hydrophilicity of natural fillers. A drop of water was placed on each of the samples, and then an image was immediately taken and the contact angle was determined. The results are shown in Figure 5. When combining the components in polymer composites, an extremely important factor is the wetting of the filler surface by the non-polar elastomer matrix (natural rubber). The hydrophobic nature of the surface of the additive contributes to a better dispersion in the polymeric media reduces the viscosity of the blend and ensures greater compatibility with rubber.

In general, natural fillers are polar in nature due to the presence of hydroxyl groups on their surface. Common nettle fits these characteristics. The measured contact angles of the surface of the leaf fillers and seeds showed a CA value of about 90°. On the other hand, samples of ground branches and roots were characterized by greater hydrophilicity, which was confirmed by the results of the measurement of the contact angle, which were respectively 80° and 72°. The relatively large contact angle can result in higher polymer–filler interactions and thus positively influence the subsequent functional properties of the composites. Differences in CA values obtained for different parts of the same plant may be due to the variable content of the individual building components of *Urtica dioica* L. Lignin is inherently more hydrophobic than cellulose [56]. Hence, it can be assumed that seeds and leaves are richer in this chemical compound. Similar conclusions were drawn on the basis of thermogravimetric analysis of the tested materials.

#### 3.1.5. The Morphology of Filler

**Figure 6:**
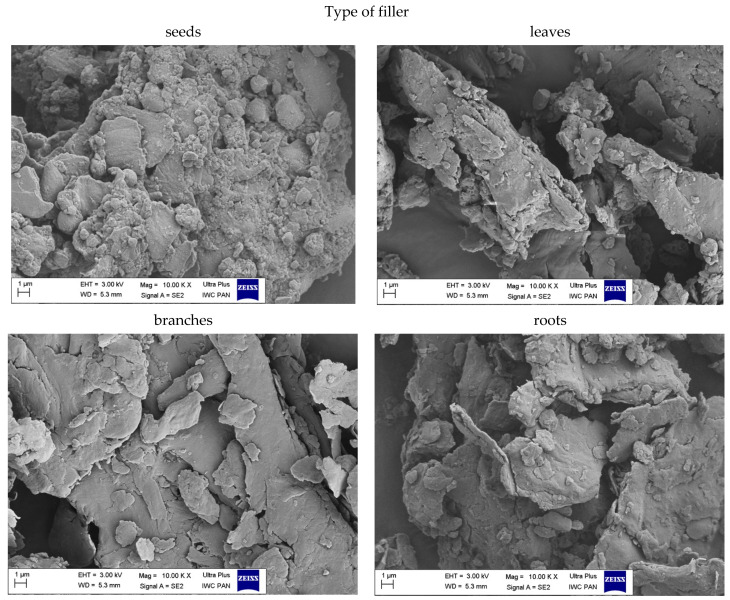
SEM images of pure common nettle: seeds (CN_s), leaves (CN_l), branches (CN_b), roots (CN_r) at the 10,000× magnification.

**Figure 7:**
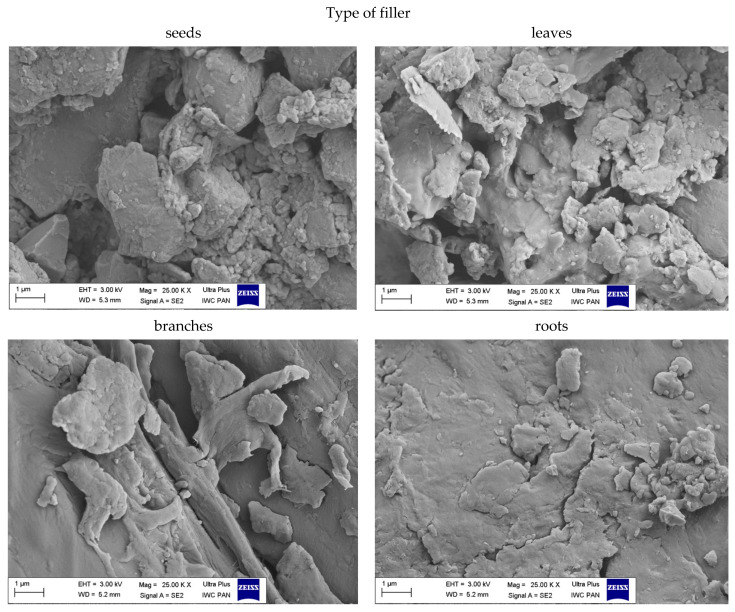
SEM images of pure common nettle: seeds (CN_s), leaves (CN_l), branches (CN_b), roots (CN_r) at the 25,000× magnification.

**Figure 8:**
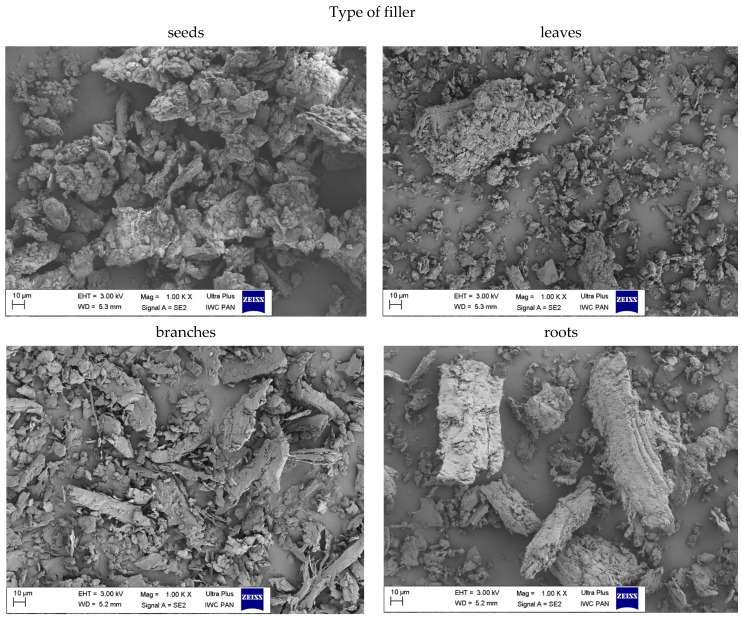
SEM images of pure common nettle: seeds (CN_s), leaves (CN_l), branches (CN_b), roots (CN_r) at the 1000× magnification.

The size and shape of the particles as well as their surface morphology are the key parameters influencing the activity of the filler in the composite material. Scanning electron microscopy images of nettle seeds, leaves, stems and roots are presented in Figure 6, Figure 7 and Figure 8.

The analysis of the presented images showed that the process of mechanical grinding caused a significant fragmentation of plant tissues. All materials had different particle sizes. There were particles with a width and length ranging from a few to several micrometers. It is worth noting, however, that many of them were much thinner, on the order of several dozen nanometers. Moreover, it was noticeable that the particles of leaves, stems and roots took the shape of thin, flat petals of a larger size. On the other hand, the seeds showed the spatial nature of their structure. SEM images at 25,000× magnification for the fillers showed the presence of smaller nanoparticles in the seeds and leaves compared to the rest of the plant. According to the lower magnification of the SEM images (Figure 8), ground common nettle particles are characterized by a high variety of shapes and sizes.

### 3.2. Characterization of Composites

#### 3.2.1. Rheological Properties of Rubber Mixtures

**Figure 9:**
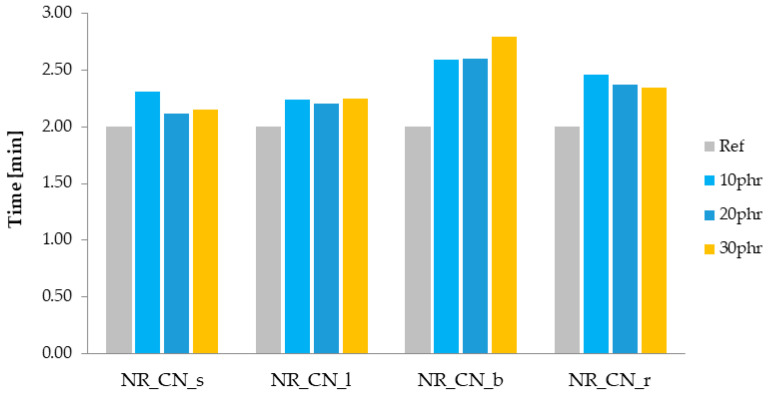
Optimal vulcanization time t_90_ (min).

**Figure 10:**
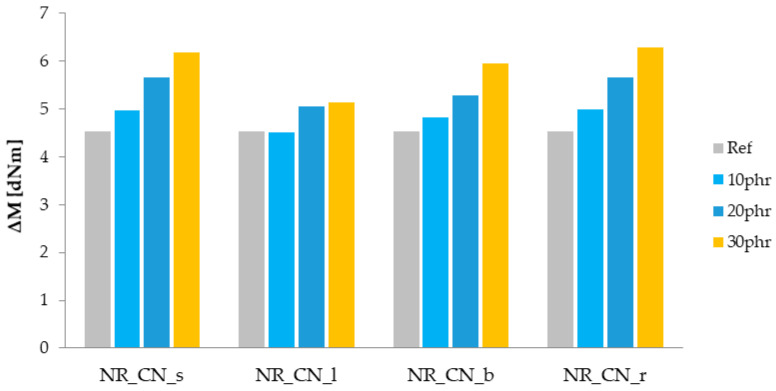
Increase in torque ΔM (dNm).

The determination of the rheometric curve is one of the basic tests that allows for determining the optimal vulcanization time (t_90_) of elastomer composites. It is the time needed to cross-link the sample under the assumed conditions in order to obtain optimal performance properties. The t_90_ value is the time when the sample reaches 90% of the maximum torque during rheometric tests. Another parameter determined from the rheometric curve is the increase in torque (ΔM), which allows for indirectly determining the cross-linking density of the tested sample [57,58]. The values of t_90_ and ΔM for natural rubber mixtures filled with nettle are presented in Figure 9 and Figure 10.

The addition of a natural filler, regardless of the material type and its amount in the biocomposite, extended the optimal vulcanization time of elastomer mixtures compared to the reference sample. This could be due to the decreased dispersion of components in elastomer matrix. Moreover, the addition of common nettle to the rubber mixture could result in absorption of crosslinking system compounds by the fillers’ particles. This might have caused a reduced effectiveness of the vulcanization process and extended the t_90_ parameter. The use of leaves and seeds had a slight effect on increasing the curing time, while the addition of roots and stems was more pronounced. The longest optimal vulcanization time (t_90_ = 2.80 min) was recorded for natural rubber filled with nettle branches.

The linear increase of the ΔM parameter with the increase in the filler content in elastomeric biocomposites in relation to the reference sample is clearly noticeable. This may be due to the addition of the non-deforming phase of the fillers, which resulted in a hydrodynamic effect. Moreover, the observed increase in ΔM is probably related to an increase in the cross-linking density of biocomposites, which in combination with the hydrodynamic effect may improve the mechanical properties.

#### 3.2.2. Payne Effect of NR Vulcanizates

The addition of a filler to the elastomeric medium causes interactions of the polymer–filler and filler–filler type. The occurrence and strength of these interactions are determined by the activity of the filler, its specific surface, size, particle morphology and content. The secondary structure is described by the Payne effect, which is defined as a decrease in the storage modulus with an increase in the strain amplitude in filled rubbers subjected to cyclic loading.

**Figure 11:**
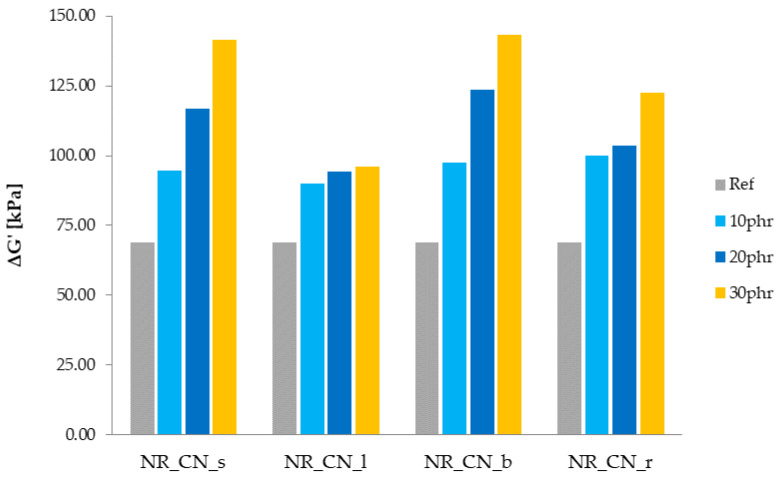
Payne effect ΔG′ (kPa) of elastomer biocomposites.

**Figure 12:**
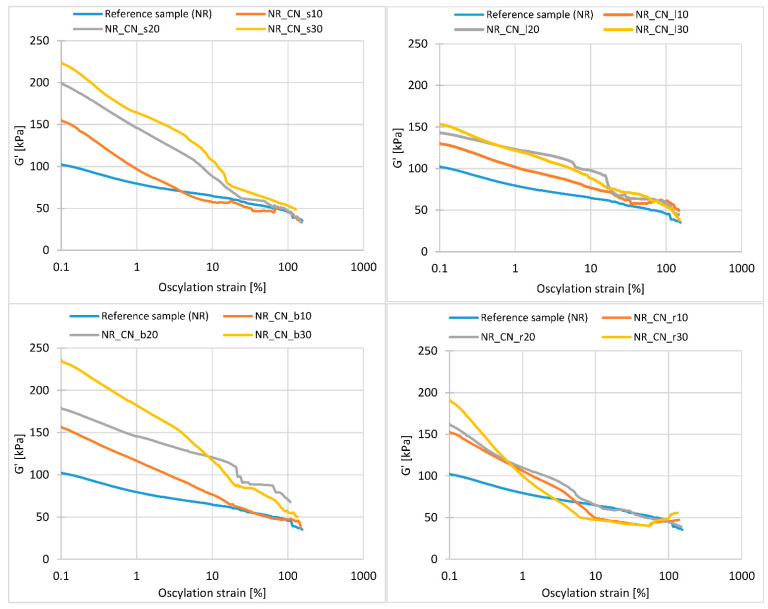
Decrease of storage modulus in the function of oscillation strain in composites filled with seeds, leaves, branches and roots.

The values of the Payne effect of natural rubber composites filled with common nettle are presented in Figure 11. The decrease of storage modulus in the function of oscillation strain is presented in Figure 12.

Regardless of the plant part, the Payne effect increased with increasing filler content. This was due to the destruction of the secondary structure of the fillers in the elastomer matrix. The greatest change in the ΔG′ parameter was observed for seeds and branches. The high value of the Payne effect may be the result of a sufficiently high activity of the fillers distributed in the polymer matrix. By creating a well-developed structure, these fillers actively strengthen the material. On the other hand, a high Payne Effect may be the result of filler clusters forming. According to obtained SEM images of fillers, the probability of agglomerates forming occurs in the case of seeds and leaves. High surface activity of the fillers increases the tendency to aggregation and agglomeration of particles. Such clusters act as stress concentrators, causing destruction of the material [59].

According to the graphs (Figure 12), the addition of nettle seeds, leaves and branches increased the storage modulus compared to the reference sample. In each case, the influence of the bioadditive content on the maximum value of G′ was observed. Moreover, the higher the filler content in the matrix, the greater the stiffness of the samples and the greater the maximum storage modulus. As the deformation energy increased, the module gradually decreased. The most intense reduction of the G′ value was observed up to a deformation of approx. 10%. This step is closely related to the destruction of the secondary structure created by the filler.

#### 3.2.3. Fourier Transform Infrared Spectroscopy (FTIR) Analysis of Composites

**Figure 13:**
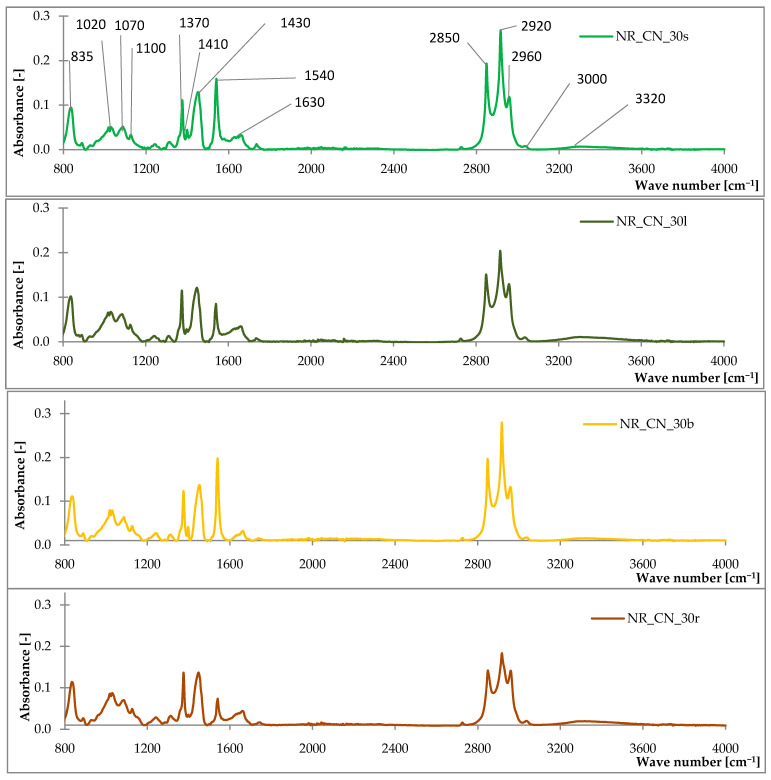
The FTIR spectra of composites filled with seeds (NR_CN_30s), leaves (NR_CN_30l), branches (NR_CN_30b), roots (NR_CN_30r).

The obtained spectra of the composites (Figure 13) showed the presence of similar bonds compared to FTIR analysis of the filler. This is confirmed by the peaks corresponding to the same wave numbers, which occurred at different intensity than in pure common nettle. The absorption peak of 835 cm^−1^ corresponds to the aliphatic group attached to the double bond C=C, which indicates the presence of natural rubber [60]. Moreover, characteristic bonds of the ν_as_ (CH_3_), ν_as_ (CH_2_) and ν_s_ (CH_2_) were recorded for the 2850–2960 cm^−1^ absorption range [61].

#### 3.2.4. Thermal Stability of Composites Determined by TGA

**Figure 14:**
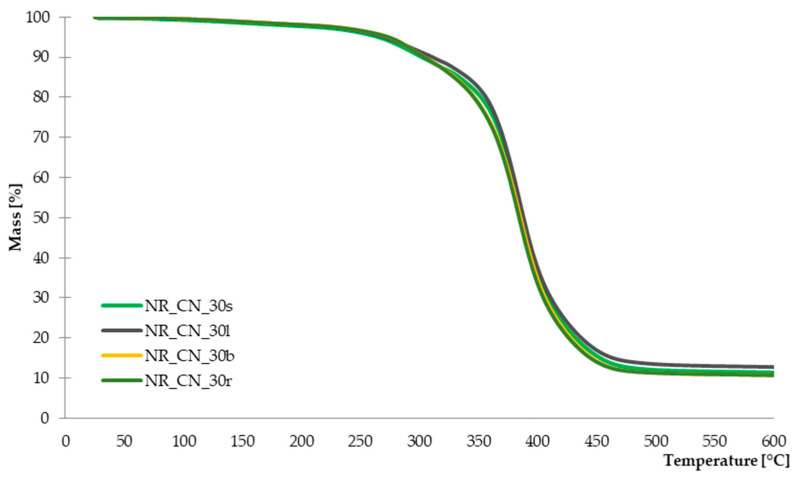
TG curves of composites filled with seeds, leaves, branches and roots.

**Figure 15:**
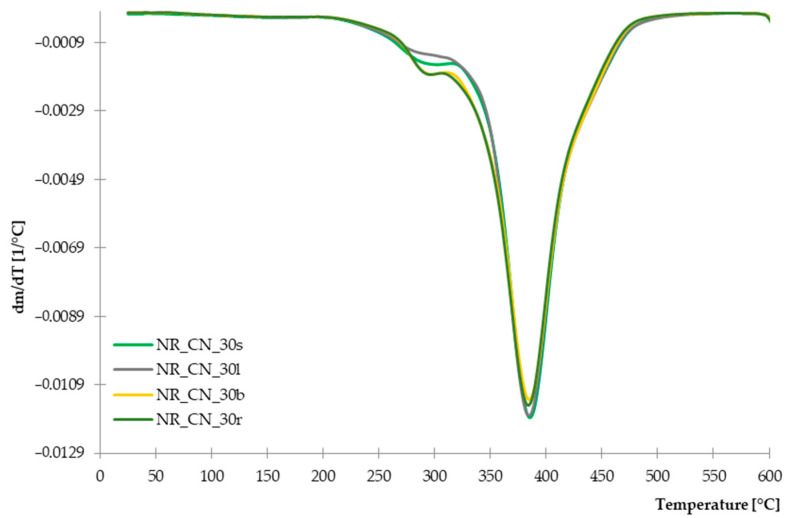
DTG curves of composites filled with seeds, leaves, branches and roots.

The recorded TG and DTG curves are presented in Figure 14 and Figure 15. The characteristic parameters of the thermal distribution are presented in Table 5.

The recorded DTG curves showed that the thermal decomposition of tested composites was a two-stage process. For all tested vulcanizates, the first stage occurred in 280 °C and lasts up to 320 °C, with a mass loss of ca. 10%. It is related to the decomposition of organic compounds from the natural filler and crosslinking system. Moreover, there was slightly lower mass loss for samples reinforced with seeds and leaves compared to the rest parts of the plant. Lower mass loss for these composites occur due to the higher lignin content of the leaves and seeds, according to the DTG curves obtained for pure common nettle parts. In addition, the total residue at 600 °C for composites filled with branches and roots was slightly lower (ca.10.7%) than the rest of vulcanizates. The greatest weight loss was recorded in the temperature range of 330–430 °C, and it is related to the decomposition of natural rubber [62] and the further degradation of natural fibers.

On the basis of the curves showed on Figure 14 and Figure 15, it can be stated that the thermal stability of composites reinforced with leaves and seeds is slightly higher compared to the rest of vulcanizates.

#### 3.2.5. Barrier Properties of the Material

**Figure 16:**
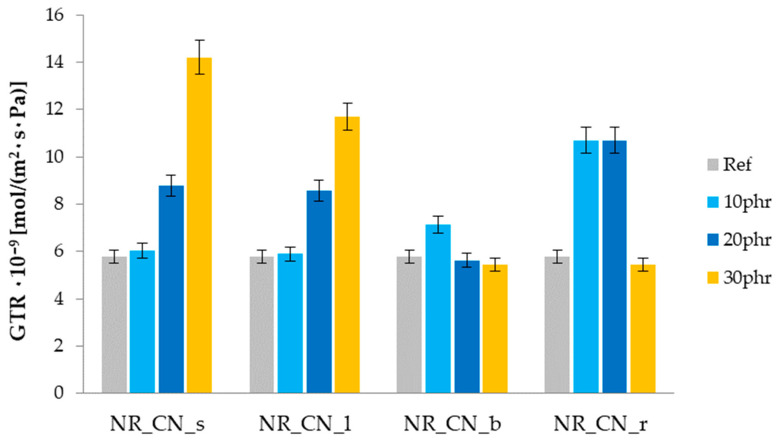
Gas transmission rate of samples.

The barrier properties of the material were defined as the ability to limit the gas (air) permeability through the surface of the biocomposite sample. The gas transfer rates (GTR) were determined. The influence of selected parts of the CN filler on the barrier properties of natural rubber is shown in Figure 16.

The type of bio-additive used had a significant impact on the barrier properties of biocomposites. Natural rubber filled with seeds and leaves of nettle showed an increase in GTR with increasing filler content. In the case of a low filling degree (10 phr), the gas flow rate through the sample was comparable to the value obtained for the unfilled system. Increasing the content of leaves and seeds caused a rapid deterioration of the barrier properties of composites. In turn, the addition of branches and roots had the opposite effect. The greater amount of such fillers inhibited gas diffusion while improving the barrier properties of materials. The differences in the limitation of air permeability could result from the variable morphology of the surface obtained for the selected parts of the plant. The plate structure of the ground roots and branches could improve the barrier properties. Thin layers of this type of fillers can act like aluminosilicates, creating a “maze” for penetrating gas [63].

#### 3.2.6. Cross-Linking Density of Biocomposites before and after Aging Processes

An important parameter influencing the functional properties of elastomeric materials is the spatial network produced by the vulcanization process and the activity of the filler. The cross-linking density of the vulcanizates containing seeds, leaves, branches and roots of *Urtica dioica* L. (Ref) is presented in Table 6. Moreover, these composites were subjected to simulated thermo-oxidative (Therm) and ultraviolet (UV) aging processes. The changes caused by the influence of external factors on the rubber vulcanizates were assessed in terms of the structure of the spatial network (Table 6).

Elastomeric biocomposites filled with lignocellulosic material derived from various parts of the common nettle showed higher cross-linking density compared to the unfilled system. The increase in the bio-additive content resulted in obtaining a more developed spatial structure. The values of the ν_e_ parameter are slightly lower for vulcanizates containing leaves compared to other composites. These results correlate with the previously discussed rheometric properties, and more specifically with the values obtained for the increase in torque (ΔM), which is considered an intermediate measure of cross-linking density.

Undoubtedly, the simulation of aging processes caused significant changes in the spatial structure of biocomposites. Both thermo-oxidation conditions and ultraviolet radiation contributed to a significant increase in their cross-linking density. One form of aging is cross-linking by recombining free macro-radicals into branched structures. Therefore, the increase in cross-linking density of most NR vulcanizates was related to cross-linking processes under the influence of elevated temperature and UV radiation. In addition, the residual content of vulcanization agents could cause additional cross-linking of elastomeric materials under the influence of the elevated temperature.

**Figure 17:**
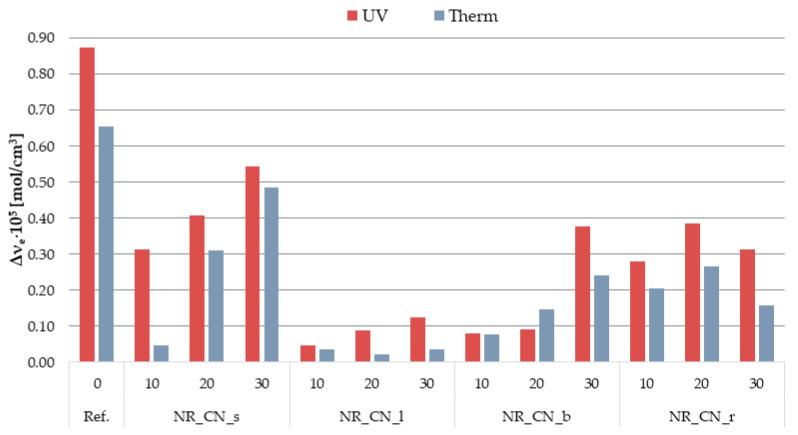
Increase in cross-linking density (Δν_e_) after thermo-oxidative (Therm) and ultraviolet (UV) aging processes with respect to unaged samples (Ref).

When analyzing the changes in the value of ν_e_ for composites after aging, it was observed that the greatest increase in this value occurred for the unfilled system (Figure 17).

Composites containing natural fillers in the form of ground parts of common nettle showed a lower increase in cross-linking density compared to the reference sample. The smallest changes in the concentration of effective nodes of the network were recorded for vulcanizates filled with leaves and branches. Perhaps, it was the effect of substances with antioxidant activity contained in the nettle. Flavonoids, phenolic acids and other polyphenols as antioxidants can react with the free radicals of macromolecules, preventing undesirable aging processes. The high antioxidant activity of the leaves was confirmed in many of the previously described studies [64,65]. In addition, all the additives used can act as UV radiation absorbers, which was confirmed by UV-Vis spectroscopy analysis.

Comparing the changes in the spatial structure of vulcanizates caused by various types of degradation processes, it was noted that UV aging resulted in higher cross-linking density values compared to the parameters obtained for biocomposites aged in thermo-oxidative conditions. Perhaps as a result of prolonged exposure to elevated temperature, apart from cross-linking processes, the degradation of the elastomeric material also took place.

#### 3.2.7. Mechanical Properties of Biocomposites before and after Aging Processes

Examination of mechanical properties provides knowledge about the strength and stress resistance of the material. The comparison of tensile strength and elongation at brake before and after aging of composites allows for indirectly predicting the lifetime of products and their possible application. Moreover, in addition, the K aging factor was measured, which determines changes in mechanical properties under the influence of unfavorable conditions to which materials are exposed during operation.

**Figure 18:**
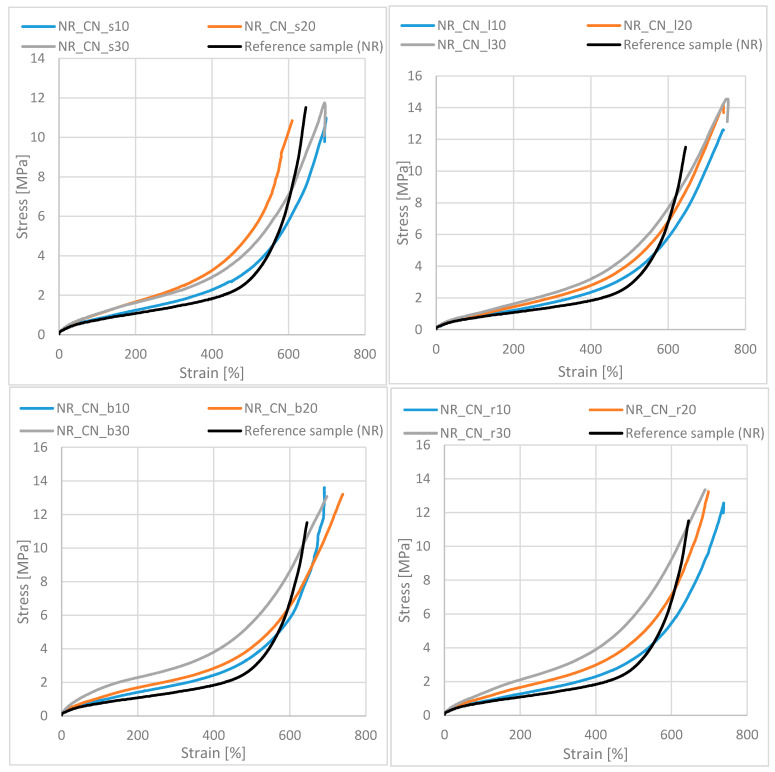
Stress–strain curves of biocomposites filled with common nettle particles.

The addition of common nettle to natural rubber improved the tensile strength of all types of biocomposites (Table 7 and Figure 18).

The stress–strain curves of vulcanizates allow for determining changes taking place in samples under the influence of uniaxial, static stretching. In each case, the filled composites showed an increase in stress compared to the reference sample at less than 500% strain. With these deformations, during stretching, the cross-links formed as a result of vulcanization were broken. On the other hand, in the case of biocomposites, an important role was played by additional nodes of the network (polymer–filler interactions), which strengthened the material causing an increase in stress. After exceeding the limit of 500–600%, the course of the curve was definitely more rapid and the stress increased more intensively. In this respect, the intermolecular interactions of the rubber, which are much stronger, were destroyed. Hence, a sudden increase in stress was observed. It is also worth noting that, in the case of the reference test, where there was no active reinforcement by the filler, the inflection in the graphs occurred at lower deformations, which consequently led to an earlier break.

The greatest strengthening effect (TS ca.14 MPa) was obtained for leaves with a filler content above 10 phr. The lowest tensile strength and elongation at break were recorded for samples with seeds. Mechanical strength of branches and roots slightly depended on the common nettle content in the vulcanizate, which was confirmed by the results of TS and Eb. The tensile strength for these composites was approx. 13 MPa.

Young’s modulus values of composites filled with various parts of common nettle were higher compared to the unfilled natural rubber sample. This is the effect of the increased stiffness of the material as well as its strengthening by the filler particles. In general, the higher the filler content in the composite, the higher the Young’s modulus value for the samples. Composites containing branches and roots showed the greatest strengthening effect in the range of low static strains (linear strain–stress relationship).

Analyzing the results of the mechanical properties of natural rubber vulcanizates after thermo-oxidative aging, the TS and Eb values decreased in almost all cases. This was most likely due to the degradation of the elastomeric material by increasing the stiffness and brittleness of the vulcanizates. As a consequence, their tensile strength and elongation at break decreased (Table 8).

**Figure 19:**
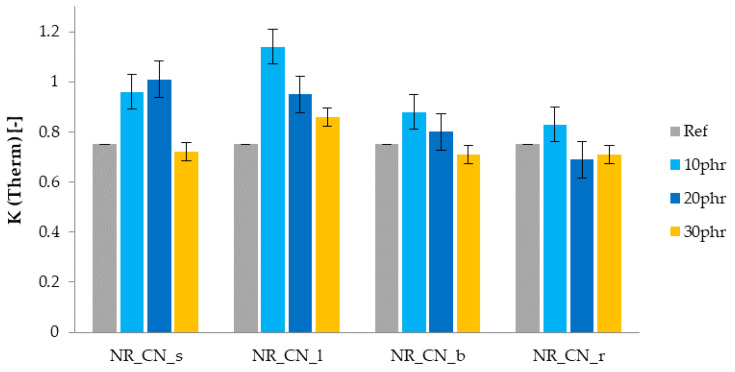
Aging factor K after thermo-oxidative aging.

The thermal aging coefficients K(Therm) were calculated from the ratio of the deformation energy of the sample before aging and after aging (Figure 19). It was found that the unfilled vulcanizate and the vulcanizates with the highest filler content (30 phr) had the lowest aging factor and thus resistance to thermo-oxidative degradation. K coefficient values close to unity mean the smallest changes in the mechanical properties of biocomposites. They were characteristic of samples containing common nettle leaves, which confirmed their best anti-aging properties.

**Figure 20:**
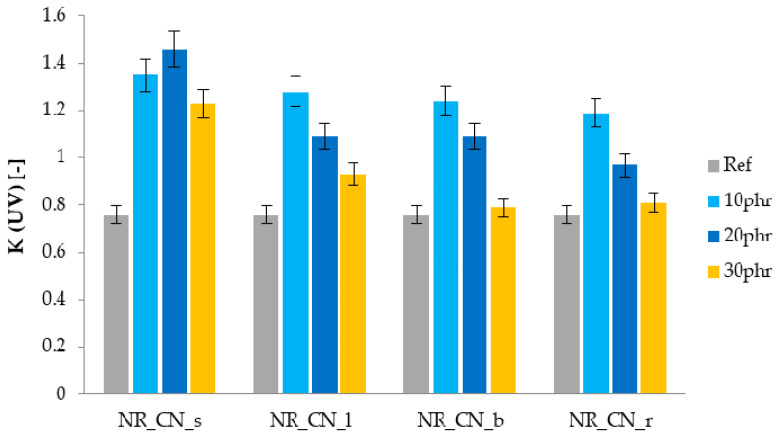
Aging factor K after UV aging.

The results of mechanical properties of NR composites after the simulation of UV aging processes are presented in Table 9. On their basis, it was observed that the reference natural rubber vulcanizate was partially degraded under the influence of UV radiation, which was confirmed by the decrease in TS and Eb values recorded after aging. A slight deterioration of the strength properties was also noted for biocomposites containing 30 phr of the filler. The remaining composites showed an increase in mechanical parameters. Consequently, the value of the K-factor for these materials was over 1 (Figure 20). In the case of UV aging, the processes taking place in the sample contributed to a slight increase in cross-linking and strengthen the sample. Moreover, the added bio-filler showed a stabilizing effect.

#### 3.2.8. Color Change

**Figure 21:**
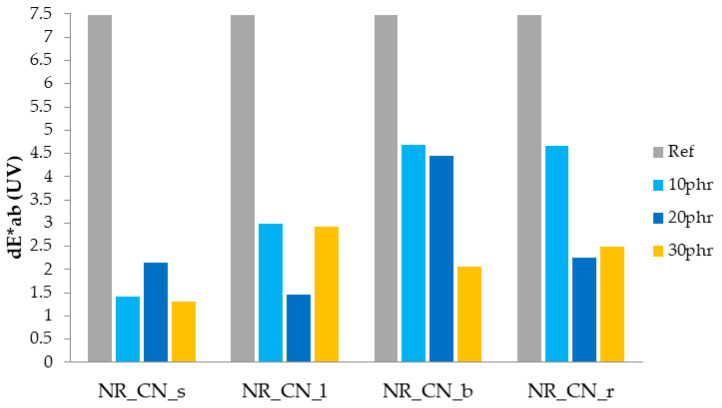
Parameters dE*ab after UV aging.

**Figure 22:**
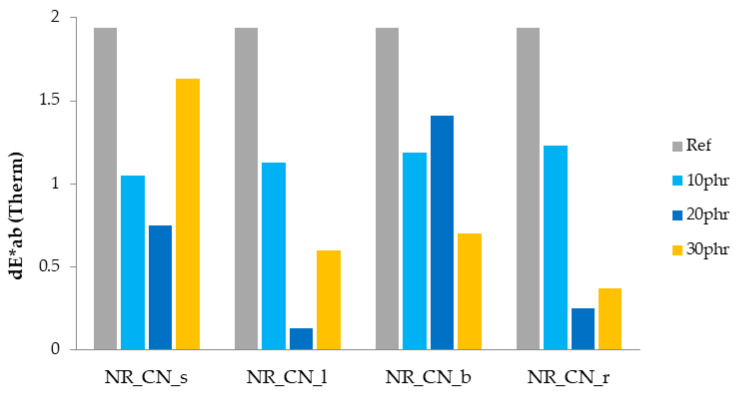
Parameters dE*ab after thermo-oxidative aging.

Color change is one of the parameters helpful in defining the aging process, as the color of the composites changes noticeably during their lifetime. The color change parameter (dE*_ab_) after UV and thermo-oxidative aging is presented on Figure 21 and Figure 22.

The color change above the value of 3 is claimed to be seen by eyes and can be related to rapid degradation processes in samples. The application of individual parts of the nettle to natural rubber not only changed their functional properties, but also, due to the presence of dyes, influenced physical properties such as color. Colorimetric measurements show that the color of elastomeric materials changed significantly after each aging process. Both the action of UV radiation and elevated temperature significantly changed the physical properties of natural rubber. The reference natural rubber vulcanizate was characterized by the lowest color stability as a result of blasting agents. In the case of composites containing common nettle as filler, the most changed color after thermal aging was observed in vulcanizates containing branches and roots. Similar observations were recorded for samples degraded by ultraviolet radiation. On the basis of the obtained colorimetric results, it can also be observed that UV radiation caused a greater color change than the increased temperature. This is due to the presence of natural stabilizers and antioxidants in common nettle, such as carotenoids and flavonoids, that are less UV resistant [38,64,65].

#### 3.2.9. Scanning Electron Microscopy of Composites

**Figure 23:**
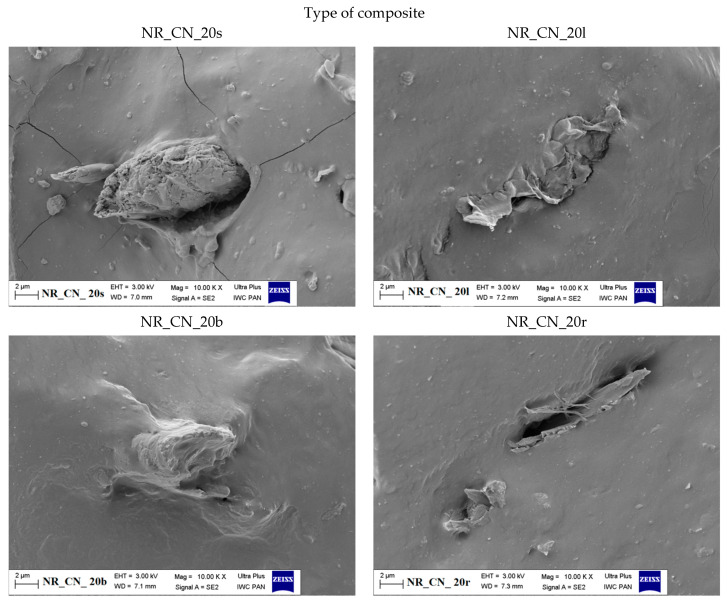
SEM images of natural rubber composites filled with 20 phr of *Urtica dioica* L.: seeds (NR_CN_20s), leaves (NR_CN_20l), branches (NR_CN_20b), roots (NR_CN_20r) at the 10,000× magnification.

**Figure 24:**
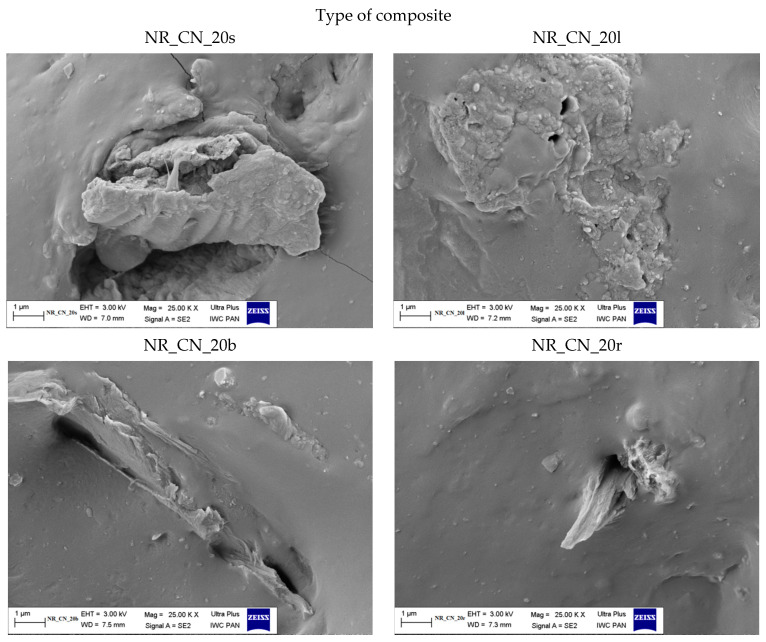
SEM images of natural rubber composites filled with 20 phr of *Urtica dioica* L.: seeds (NR_CN_s), leaves (NR_CN_l), branches (NR_CN_b), roots (NR_CN_r) at the 25,000× magnification.

The morphology of biocomposites containing seeds, leaves, branches and roots of the common nettle is presented in Figure 23 and Figure 24.

In the composite containing seeds and leaves, it can be seen that the particles were filled both in the form of well-dispersed nanometric elements and slightly larger clusters, constituting larger primary particles or their agglomerates. In the case of the remaining vulcanizates, the fillers took the form of thin plate structures. The adhesion of the bioadditive particles to the elastomer matrix was varied. In general, the surface of the particles adhered closely to the rubber, but there were also gaps between the polymer and the filler. Such an arrangement of the fillers could influence the strengthening of the elastomeric material in various ways. On the one hand, good adhesion of the filler to the matrix can improve the mechanical properties; on the other hand, the agglomerates formed can act as stress concentrating elements leading to local surface destruction.

## 4. Conclusions

In this research, the characteristic of *Urtica dioica* L. was presented to determine the effectiveness of its functional compounds for the modification for a natural rubber composite. The FTIR spectra analysis of each plant part showed that it is mainly composed of lignocellulosic material, which was confirmed by obtained TG and DTG curves. Moreover, it was observed that the seeds and leaves contain more thermally less stable lignin and hemicellulose than branches and roots. The results of UV-Vis study on common nettle showed the presence of chlorophyll, carotenoids such as lutein, lutein isomers, β-carotene and β-carotene isomers and high antioxidant: flavonol glycosides and phenolic acids. The contact angle (CA) measurement confirmed polar and hydrophilic characteristics of the natural filler. Differences in CA values and particle size or shape presented on SEM images for different parts of the common nettle can be stated as a result of the variable content of the building components.

The addition of *Urtica dioica* L. in natural rubber composite extended optimal vulcanization time (t_90_) compared to the reference sample, especially in the case of roots and stems. Moreover, the ΔM parameter values increased linear with the increase in the filler content. Linear increase was also registered in the case of Payne effect for all parts of the plant. It can be stated that relatively high values of ΔG′ parameter are a result of a sufficiently high activity of the fillers distributed in the polymer matrix or may be caused by formation of clusters.

According to the thermogravimetric analysis, thermal stability of composites reinforced with leaves and seeds was slightly higher compared to the rest of vulcanizates.

The improvement in barrier properties of natural rubber composite reinforced with common nettle was observed based on the lowest gas transmission rate for vulcanizates with the highest content (30 phr) of branches and roots. An increase in cross-linking density was observed for all vulcanizates filled with common nettle regardless of the part of the plant. The analysis of changes in the value of ν_e_ after ultraviolet and thermo-oxidative aging showed that composites with addition of natural filler are characterized by lower cross-linking density compared to the reference sample.

On the basis of the examinated TS and Eb values, it can be stated that the addition of *Urtica dioca* L. increases the mechanical strength of natural rubber biocomposites with the greatest strengthening effect for leaves above 10 phr filler content. The decreasing tendency of mechanical properties after thermo-oxidative aging was observed in most of the examined samples. On the other hand, after UV aging, the slight increase in TS and Eb parameters was noticed. The lowest values of aging coefficients K were observed for unfilled vulcanizates after both types of simulated aging processes. The changes in the properties of aged biocomposites were also confirmed by the results of the color change test. According to the obtained SEM images, the best adhesion to the elastomer matrix occurred for leaves and branches of common nettle.

Conducted research provides information about the impact of *Urtica dioica* L. on natural rubber composites, which can find its potential application in protective materials such as external coatings, housings or lining. Moreover, it enables to extend studies of natural fillers in the field of elastomeric composites.

## Figures and Tables

**Table 1:** Compositions of elastomeric biocomposites.

Sample Name	CN	NR	Stearin	ZnO	MBT	Sulphur
	(phr ^1^)					
Reference Sample (NR)	0	100	1	5	2	2
NR_CN_s10	10	100	1	5	2	2
NR_CN_s20	20	100	1	5	2	2
NR_CN_s30	30	100	1	5	2	2
NR_CN_l10	10	100	1	5	2	2
NR_CN_l20	20	100	1	5	2	2
NR_CN_l30	30	100	1	5	2	2
NR_CN_b10	10	100	1	5	2	2
NR_CN_b20	20	100	1	5	2	2
NR_CN_b30	30	100	1	5	2	2
NR_CN_r10	10	100	1	5	2	2
NR_CN_r20	20	100	1	5	2	2
NR_CN_r30	30	100	1	5	2	2

^1^ phr—parts per hundred parts of rubber.

**Table 2:** Sieve analysis of common nettle.

Fraction [mm]	Sieve Sizes [mm]	Seeds		Leaves		Branches		Roots	
		(g)	(%)	(g)	(%)	(g)	(%)	(g)	(%)
0.500–1.00	0.500	0.45	1.13	1.4	2.34	2.66	4.44	1.65	4.13
0.250–0.500	0.250	7.60	19.01	12.74	21.23	13.93	23.21	9.20	23.01
0.125–0.250	0.125	23.82	59.55	35.07	58.45	34.04	56.74	22.66	56.65
0.065–0.125	0.065	8.12	20.31	10.79	17.98	9.37	15.61	6.48	16.21
	Σ	40	100	60	100	60	100	40	100

**Table 3:** Characteristic bonds of the FT-IR spectra of the common nettle samples studied.

Peak Assignments	Type of Vibration	Absorption Ranges [cm^−1^]
O-H hydrogen	stretching	3760
O-H phenols & alcohols	stretching	3650–3200
C-H vinyl & acrylic	stretching	3100–3010
C-H aliphatic	stretching	2970–2800
C=O	stretching	1730–1690
C=N	stretching	1750–1500
C=C alkenes	stretching	1680–1610
C=C aryl	stretching	1600–1500
C-C aliphatic	stretching	1500–600
C=C aromatic;	skeletal vibration	1441
C-H aliphatic	deformation	1370–1340
C-N	stretching	1360–1180
C-O, C-C, -C-O-C-	asymmetric & symmetric stretching	1280–1150
C-O-C	axial deformation	1060
C-H vinyl	deformation	995–675
C-H aryl	glucose ring stretching/out of plane deformation	900–690

**Table 4:** Thermal stability of fillers determined by TGA.

Sample Name	T_10_^1^ (°C)	Δm_100_^2^ (%)	Δm_335_^3^ (%)	Residue at 600 °C ^4^ (%)
CN_s	225.02	3.04	55.76	31.86
CN_l	226.40	2.09	59.31	36.34
CN_b	237.40	2.78	48.13	24.88
CN_r	249.23	2.79	50.39	25.60

^1^ T_10_—temperature of 10% mass loss, ^2^ Δm_100_—mass loss at 100 °C of the sample, ^3^ Δm_335_—mass loss at 335 °C, ^4^ Residue at 600 °C—mass of the sample at 600 °C.

**Table 5:** Thermal stability of composites determined by TGA.

Sample Name	T_10_^1^ (°C)	Δm_385_^2^ (%)	Residue at 600 °C ^3^ (%)
NR_CN_30s	303.00	46.98	11.39
NR_CN_30l	311.00	45.93	12.76
NR_CN_30b	304.00	49.33	10.72
NR_CN_30r	304.00	50.65	10.71

^1^ T_10_—temperature of 10% mass loss, ^2^ Δm_385_—mass loss at 385 °C, ^3^ Residue at 600 °C—mass of the sample at 600 °C.

**Table 6:** Results of cross-linking density.

Sample Name	Filler Content (phr)	ν_e_ × 10^5^ (mol/cm^3^)		
		Ref	UV	Therm
Ref. Sample (NR)	0	1.78 ± 0.03	2.66 ± 0.02	2.44 ± 0.03
NR_CN_s	10	2.11 ± 0.03	2.43 ± 0.04	2.16 ± 0.04
	20	2.14 ± 0.03	2.55 ± 0.04	2.45 ± 0.04
	30	2.25 ± 0.01	2.80 ± 0.05	2.74 ± 0.05
NR_CN_l	10	1.97 ± 0.02	2.02 ± 0.02	2.01 ± 0.02
	20	2.03 ± 0.03	2.11 ± 0.02	2.05 ± 0.03
	30	2.04 ± 0.02	2.16 ± 0.02	2.08 ± 0.04
NR_CN_b	10	2.18 ± 0.02	2.26 ± 0.02	2.25 ± 0.02
	20	2.22 ± 0.02	2.31 ± 0.03	2.37 ± 0.01
	30	2.40 ± 0.03	2.78 ± 0.04	2.65 ± 0.02
NR_CN_r	10	2.07 ± 0.03	2.35 ± 0.04	2.27 ± 0.02
	20	2.12 ± 0.01	2.50 ± 0.02	2.38 ± 0.02
	30	2.53 ± 0.03	2.84 ± 0.03	2.69 ± 0.13

**Table 7:** Tensile strength (MPa), Young Modulus (MPa) and elongation at brake (%) of biocomposites.

Sample Name	Filler Content (phr)	Young Modulus (MPa)	TS (MPa)	Eb (%)
Ref. Sample (NR)	0	0.187 ± 0.002	11.48 ± 0.05	640.05 ± 2.80
NR_CN_s	10	0.193 ± 0.002	12.17 ± 0.64	677.80 ± 10.83
	20	0.204 ± 0.002	11.90 ± 0.12	645.83 ± 4.91
	30	0.216 ± 0.002	11.70 ± 0.41	679.85 ± 14.64
NR_CN_l	10	0.200 ± 0.002	12.41 ± 0.28	697.4 ± 21.60
	20	0.198 ± 0.002	14.13 ± 0.10	723.27 ± 8.54
	30	0.213 ± 0.002	14.22 ± 0.65	744.43 ± 8.45
NR_CN_b	10	0.205 ± 0.002	13.42 ± 0.70	679.16 ± 13.05
	20	0.209 ± 0.002	13.18 ± 0.12	733.04 ± 8.28
	30	0.285 ± 0.002	13.23 ± 0.22	718.16 ± 14.93
NR_CN_r	10	0.201 ± 0.002	13.06 ± 0.58	738.07 ± 1.00
	20	0.214 ± 0.002	13.25 ± 1.17	700.51 ± 13.36
	30	0.247 ± 0.002	13.41 ± 0.41	713.07 ± 23.36

**Table 8:** Tensile strength (MPa) and elongation at brake (%) of biocomposites after thermooxidative aging.

Sample Name (Therm)	Filler Content (phr)	TS (MPa)	Eb (%)
Ref. Sample (NR)	0	10.15 ± 0.05	543.17 ± 3.04
NR_CN_s	10	12.05 ± 1.91	657.28 ± 7.89
	20	12.37 ± 0.28	625.02 ± 0.71
	30	9.34 ± 0.10	614.94 ± 18.98
NR_CN_l	10	13.57 ± 0.26	726.70 ± 9.91
	20	13.49 ± 0.44	716.44 ± 0.68
	30	13.13 ± 0.09	691.45 ± 12.30
NR_CN_b	10	11.96 ± 0.76	673.90 ± 4.61
	20	11.42 ± 0.64	675.95 ± 5.25
	30	10.08 ± 0.59	665.38 ± 13.81
NR_CN_r	10	11.98 ± 2.86	665.91 ± 3.19
	20	10.18 ± 1.93	628.26 ± 14.76
	30	10.58 ± 0.01	643.15 ± 2.62

**Table 9:** Tensile strength (MPa) and elongation at brake (%) of biocomposites after UV aging.

Sample Name	Filler Content (phr)	TS (MPa)	Eb (%)
Ref. Sample (NR)	0	9.65 ± 0.58	578.38 ± 3.75
NR_CN_s	10	14.60 ± 0.46	761.47 ± 9.60
	20	15.00 ± 0.51	750.37 ± 8.24
	30	13.55 ± 0.13	724.66 ± 3.02
NR_CN_l	10	13.68 ± 0.02	809.49 ± 1.89
	20	14.29 ± 0.87	781.61 ± 6.55
	30	12.95 ± 0.32	757.11 ± 5.53
NR_CN_b	10	14.33 ± 0.69	788.07 ± 9.62
	20	13.81 ± 0.25	765.25 ± 3.26
	30	10.71 ± 0.06	704.45 ± 3.26
NR_CN_r	10	14.75 ± 0.13	776.27 ± 5.73
	20	12.52 ± 0.58	717.73 ± 8.26
	30	11.55 ± 0.63	668.58 ± 8.45

## Data Availability

Data sharing not applicable.
